# Different Primary Sites of Hypopharyngeal Cancer Have Different Lymph Node Metastasis Patterns: A Retrospective Analysis From Multi-Center Data

**DOI:** 10.3389/fonc.2021.727991

**Published:** 2021-09-20

**Authors:** Xiwei Zhang, Ye Zhang, Xiaoduo Yu, Ying Sun, Susheng Miao, Shaoyan Liu, Zhengjiang Li, Junlin Yi, Changming An

**Affiliations:** ^1^Department of Head and Neck Surgery, National Cancer Center/National Clinical Research Center for Cancer/Cancer Hospital, Chinese Academy of Medical Sciences and Peking Union Medical College, Beijing, China; ^2^Departments of Radiation Oncology, National Cancer Center/National Clinical Research Center for Cancer/Cancer Hospital, Chinese Academy of Medical Sciences and Peking Union Medical College, Beijing, China; ^3^Departments of Radiation, National Cancer Center/National Clinical Research Center for Cancer/Cancer Hospital, Chinese Academy of Medical Sciences and Peking Union Medical College, Beijing, China; ^4^State Key Laboratory of Oncology in South China, Collaborative Innovation Center of Cancer Medicine, Guangdong Key Laboratory of Nasopharyngeal Carcinoma Diagnosis and Therapy, Sun Yat-sen University Cancer Center, Guangzhou, China; ^5^Department of Radiation Oncology, Harbin Medical University Cancer Hospital, Harbin, China

**Keywords:** hypopharyngeal cancer (HPC), lymph node metastasis (LNM), magnetic resonance imaging (MRI), retropharyngeal lymph nodes (RPLN), pattern, bilateral

## Abstract

**Background:**

Most hypopharyngeal cancers (HPCs) develop lymph node metastasis (LNM) at initial diagnosis. Understanding the pattern of LNM in HPC could help both surgeons and radiologists make decisions in the management of cervical lymph nodes.

**Methods:**

A total of 244 newly diagnosed HPC patients between January 2010 and December 2018 were recruited from three specialized cancer hospitals in mainland China. All patients received pre-treatment magnetic resonance imaging (MRI), and definitive radiotherapy with or without concurrent chemotherapy. We reassessed the features of the primary tumor (tumor size, primary location, and extent of invasion) and the involvement of lymph nodes at each level. According to the incidence of LNM, these levels were sequenced and sorted into drainage stations. Univariate and multivariate analyses were used to determine the risk factors for bilateral and regional lymph node metastasis.

**Results:**

The cohort consisted of 195 piriform sinus cancers (PSC), 47 posterior wall cancers (PWC), and 2 post-cricoid cancers (PCC). A total of 176 patients (72.1%) presented with MRI-detectable LNMs. The overall LNM rates for level II-VI and retropharyngeal lymph nodes (RPLNs) were 59.0%, 52.9%, 14.3%, 1.6%, 2.9%, and 16.4%, respectively. Based on the prevalence of LNM at each level, we hypothesize that the lymphatic drainage of PSC was carried out in sequence along three stations: Level II and III (61.0% and 55.4%), Level IV and RPLN (15.9% and 11.3%), and Level V and VI (1.5% and 3.1%). For PWCs, lymphatic drainage is carried out at two stations: Level II, III, and RPLN (48.9%, 40.4%, and 34.0%) and Level IV-VI (6.4%, 0%, and 2.1%). According to univariate and multivariate analyses, posterior wall invasion was significantly correlated with bilateral LNM (P = 0.030, HR = 2.853 95%CI, 1.110-7.338) and RPLN metastasis (P = 0.017, HR = 2.880 95%CI, 1.209-6.862). However, pyriform sinus invasion was less likely to present with bilateral LNM (P = 0.027, HR = 0.311, 95%CI, 0.111-0.875) and RPLN metastasis (P = 0.028, HR = 0.346, 95%CI, 0.134-0.891).

**Conclusions and Relevance:**

The primary tumor site and extent of invasion are related to the pattern of lymph node metastasis. That is, the metastasis would drainage station by station along different directions.

## Introduction

The hypopharynx, which connects the oropharynx, larynx, and cervical esophagus, is the junction of the upper respiratory and digestive tracks. Hypopharyngeal cancer (HPC) is dominated by squamous cell cancer and accounts for only 6% of all head and neck cancers (1). The prognosis of HPC is relatively poor compared with that of other head and neck cancers, with a 5-year overall survival of only 30%–35% (2, 3).

Due to the lack of obvious symptoms, most HPC patients have developed a progressive disease at their initial diagnosis, with lymph node metastasis (LNM) incidence as high as 60% (4). Therefore, management of lymph nodes must be considered in the treatment planning of most patients with HPC. Since lymphatic drainage of the hypopharynx is abundant, the primary tumors may spread along different paths to the lateral neck or posteriorly to the posterior wall. Understanding the pattern of lymph node metastasis in HPC and the relationship between the primary tumor and LNM could help both surgeons and radiologists make decisions in cervical lymph node management.

Magnetic resonance imaging (MRI), with a higher ability for detailed presentation of soft tissue, is superior to computed tomography (CT) in the evaluation of cervical lymph node involvement. Here, we collected pre-treatment MRIs of HPC from three cancer centers in mainland China for review and aimed to determine the pattern of nodal spread and the correlation between the features of the primary tumor and LNM.

## Materials and Methods

Patients with pathologically proven hypopharyngeal squamous cell cancer from three specialized cancer hospitals in mainland China (Cancer Hospital, Chinese Academy of Medical Sciences; Sun Yat-sen University Cancer Center; Harbin Medical University Cancer Hospital) between January 2010 and December 2018 were recruited. All patients received definitive radiotherapy (GTV ≥ 66Gy) with or without concurrent chemotherapy, and cervical MRI was performed before treatment. Unavailable pre-treatment MRI, distant metastasis before initial treatment, and second primary cancer were the exclusion criteria. This study was approved by the institutional ethics committee of Cancer Hospital, Chinese Academy of Medical Sciences (NCC2018J-004). Informed consent was obtained before surgery.

All pre-treatment MRIs were reviewed by two dedicated head and neck radiologists. The features of the primary tumor and the presence of lymph nodes at each level of the neck were reassessed. Tumor information included tumor size, tumor location, and extent of invasion. Lymph nodes were assigned according to the RGOT guidelines. Seven groups of lymph nodes, levels I-VI and retropharyngeal lymph nodes (RPLN), were assessed. The following criteria were considered as a radiographically positive LN: in the axial plane, the largest short diameter of the retropharyngeal node ≥ 5 mm, ≥ 11 mm at level II, ≥ 10 mm at other level, and any visible median RPLN; three lymph node grouping (each of which should have a minimal axial dimension of 8–10 mm); lymph node with circular enhancement or central necrosis; and lymph node with extracapsular spread (5). If MRI cannot determine the metastasis, we will evaluate it in combination with pre-treatment CT scans and ultrasound.

The prevalence of each cervical level was calculated using descriptive statistics with SPSS 25.0 (IBM Corp. Released 2017. IBM SPSS Statistics for Windows, Version 25.0. Armonk, NY: IBM Corp.). Predictors (tumor size, lesion location, and the extent of invasion) of the presence of bilateral LNM were examined using univariate and multivariate logistic regression. A two-sided p-value of less than 0.05 was considered statistically significant.

## Results

### Baseline Characteristics of Patients

Of the 244 patients, 262 (96.0%) were male, only 11 (4.0%) were female. The median age of the cohort was 56 years (range: 36–85 years). The primary anatomical site of the tumor was located at pyriform sinus for 195 patients (79.9%), posterior pharyngeal wall for 47 patients (19.3%), and postcricoid area for only 2 patients (0.8%). According to the AJCC 8th staging-system, 122 patients (50.0%) were restaged as T1-2, 122 patients (50.0%) were T3-4. N0-1 were observed in 116 patients (47.5%). N2-3 were observed in 128 patients (52.5%). Among them, 37 patients (15.2%) were of stage I-II and 207 patients (84.8%) were of stage III-IV. In our group, all patients received definitive radiotherapy (GTV ≥ 66Gy). Among them, 92 (37.7%) and 161 patients (66.0%) received induced or concurrent chemotherapy respectably. (**Table 1**) The 3-year and 5-year overall survival (OS) of our group were 49.9% and 36.4%.

**Table 1:** The baseline characteristics of the patients.

Characteristic	Patients (%)
Gender	
Male	237 (97.1)
Female	7 (2.9)
Age (y.)	
<50	63 (25.8)
≥50	181 (74.2)
Tumor location	
Pyriform sinus	195 (79.9)
Posterior pharyngeal wall	47 (19.3)
Postcricoid	2 (0.8)
T-stage	
T1-2	122 (50.0)
T3-4	122 (50.0)
N-stage	
N0-1	116 (47.5)
N2-3	128 (52.5)
TNM stage	
I-II	37 (15.2)
III-IV	207 (84.8)
Induced chemotherapy	
Yes	92 (37.7)
No	152 (62.3)
Concurrent chemoradiotherapy	
Yes	161 (66.0)
No	83 (34.0)

### LNM Incidence

In our cohort, a total of 176 patients (72.1%) presented with MRI-detectable LNM. No LNM was found in level I in any of the patients. The most common LNM regions are Levels II and III, with incidences of 59.0% and 52.9%, respectively. Level IV and RPLN followed with incidences of 14.3% and 16.4%, respectively. LNMs in Levels V and VI were rare, with an incidence of only 1.6% and 2.9%, respectively. In our cohort, 40 patients (16.4%) presented with bilateral LNMs, all of which were restricted to levels II, III, and RPLNs (**Table 2**).

**Table 2:** The incidence of lymph nodes metastasis at each level among 244 patients.

Level	Unilateral (%)	Bilateral (%)	Total (%)
I	0	0	0
II	120 (49.2)	24 (9.8)	144 (59.0)
III	121 (49.6)	8 (3.3)	129 (52.9)
IV	35 (14.3)	0	35 (14.3)
V	4 (1.6)	0	4 (1.6)
VI	7 (2.9)	0	7 (2.9)
RPLN^*^	25 (10.2)	15 (6.1)	40 (16.4)
Total (%)	136 (55.7)	40 (16.4)	176 (72.1)

*Retropharyngeal Lymph node.

### Patterns of LNM

**Figure 1:**
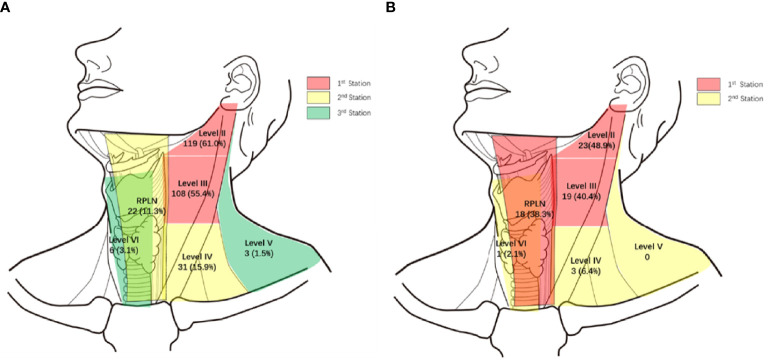
Specific distribution of LNMs. **(A)** Specific distribution of LNM in 195 piriform sinus cancers. **(B)** Specific distribution of LNMs in 47 posterior wall cancers. (%) Percent of LNMs in all 195 piriform sinus cancers or 47 posterior wall cancers.

**Figure 2:**
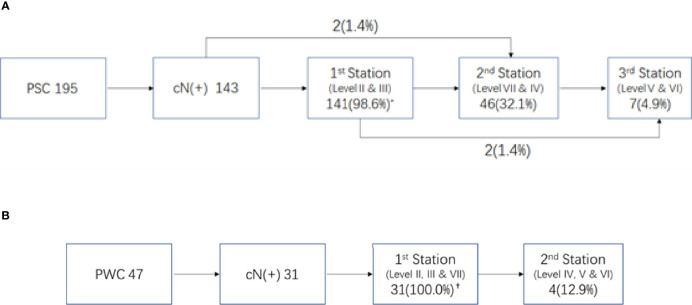
Lymphnode metastasis pattern in different primary site of hypopharyngeal cancer. **(A)** Pattern of LNM in piriform sinus cancer (PSC) * Percent of LNMs in 143 N(+) patients. **(B)** Pattern of LNM in posterior wall (PPC). ^†^Percent of LNMs in 31 N(+) patients.

Of the 195 PSC patients, 143 (73.3%) were diagnosed with cervical LNMs. More than half of the patients had LNMs in levels II and III, with metastasis rates of 61.0% and 55.4%, respectively. For Level IV and RPLNs, the LNM rates were 15.9% and 11.3%, respectively. However, LNMs were rare in Levels V and VI, with incidences of only 1.5% and 3.1%, respectively (**Figure 1A** shows a specific distribution). Based on the incidence of LNM at each level, we concluded that lymphatic drainage of PSC was carried out sequentially along three stations (See **Figure 2A**). These are Levels II and III for the 1^st^ station, Level IV and RPLN for the 2^nd^ station, and Levels V and VI for the 3^rd^ station. Based on our hypothesis, lymph node skip metastasis was rare in the cohort, as it was found in only four patients (2.8%).

**Figure 3:**
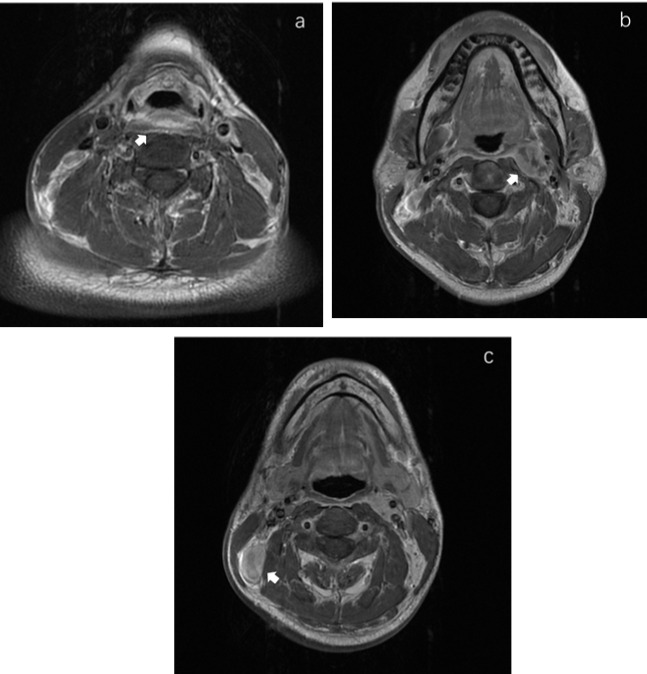
MRI images in T1 with contrast of a posterior wall cancer patient. **(A)** The primary focus of posterior wall cancer. **(B)** The left retropharyngeal lymphnode metastasis. **(C)** The metastatic lymphnode in right Level II.

Of the 47 PWCs, LNMs were detected in 31 patients (66.0%). Besides Levels II (48.9%) and III (40.4%), RPLNs (38.3%) also presented a high incidence of LNM. However, LNMs in level IV were less common than those in PSC. The LNM rates in Levels IV-VI were only 6.4%, 0%, and 2.1%, respectively. (**Figure 1B** shows specific distributions). Therefore, we hypothesize that the lymphatic drainage of PWC is carried out along two stations: Levels II, III, and RPLN as the 1^st^ station, and Levels IV-VI as the 2^nd^ station (**Figure 2B**). No lymph node skip metastasizes were found based on this hypothesis. **Figure 3** shows the T1 with contrast images of a patient with PWC (**Figure 3A**). The patient has LNMs in left RPLN (**Figure 3B**) and right Level II (**Figure 3C**).

Only two PCCs were included in our cohort. A patient was diagnosed with LNMs in ipsilateral II-IV. The other patient had LNMs in bilateral levels II and III.

### Univariate and Multivariate Analyses

To determine the risk factors for bilateral and regional LNM, univariate and multivariate analyses were used, the results of which are displayed in **Table 3**. According to univariate analysis, invasion of the posterior wall was a risk factor for bilateral LNM. Patients with pyriform sinus invasion were less likely to develop bilateral LN. Further multivariate analysis showed that posterior wall and pyriform sinus invasion were still statistically significant, with P = 0.002 (HR = 3.524 95%CI, 1.559-7.964) and P = 0.027 (HR = 0.311, 95%CI, 0.111-0.875), respectively.

**Table 3:** The risk factors for bilateral LNM and PRLN metastasis.

Factors		Univariate analysis		Multivariate analysis	
		HR (95%CI)	*p*	HR (95%CI)	*p*
Bilateral LNM	Pyriform sinus invasion	0.165 (0.064-0.430)	0.000	0.311 (0.111-0.875)	0.027
	Posterior wall invasion	4.812 (2.259-10.252)	0.000	3.524 (1.559-7.964)	0.002
PRLN metastasis	Tumor Size				
	≤2cm		0.023		
	2-4cm	6.206 (0.809-47.618)	0.079		
	>4cm	12.364 (1.561-97.907)	0.017		
	Pyriform sinus invasion	0.213 (0.095-0.481)	0.000	0.346 (0.134-0.891)	0.028
	Posterior wall invasion	4.543 (2.201-9.378)	0.000	2.471 (1.047-5.832)	0.039

Univariate analysis revealed that the presence of retropharyngeal lymph nodes was associated with larger tumor size (> 4 cm) and posterior wall invasion. However, HPCs with pyriform sinus invasion were less likely to have PRLN metastasis. However, only posterior wall invasion (P = 0.017, HR = 2.880, 95%CI, 1.209-6.862) and pyriform sinus invasion (P = 0.028, HR = 0.346, 95%CI, 0.134-0.891) were associated with PRLN metastasis in multivariate analysis.

## Discussion

Hypopharyngeal cancer is a relatively rare malignancy with poor prognosis. Due to the lack of obvious symptoms, most patients with HPC have lymph node metastasis at the initial diagnosis. Therefore, the management of lymph nodes must be considered in the treatment plan of most patients with HPC. Understanding the lymph node metastasis pattern of HPC and the relationship between primary tumor and LNM can help clinicians make decisions in the treatment of cervical lymph nodes.

Various imaging technologies, such as CT, MRI, and ^18^F-FDG, have been widely used in the evaluation of head neck cancer (6, 7). MRI has a higher ability in the presentation of soft tissue than CT or PET, especially in the evaluation of primary tumors, since the tumor may extend to the mucous membrane alone. Moreover, MRI has been shown to be superior to CT imaging for the detection of metastatic RPLNs (8). Therefore, we chose pre-treatment MRI as mandatory in identifying both the primary tumor and LNM in HPC patients in our cohort. Hypopharyngeal cancer is a relatively rare disease among head and neck tumors. Therefore, it is difficult to obtain large-sample-size image data, especially MRIs, in a single center. Here, we combined three specialized cancer hospitals and collected the pre-treatment MRIs of 244 patients.

Because of the abundant lymphatic drainage in the hypopharynx, most patients will have developed lymph node metastasis at the initial treatment. The reported LNM rate at diagnosis is 65% (5, 9, 10). Among them, the most common metastasis was located at levels II and III, followed by level IV. The LNM rate of our group is similar to that of previous reports. However, in our cohort, the incidence of RPLN metastasis was as high as 16.4%, which is equivalent to that of level IV (14.3%). This may be because MRI is superior in detecting RPLNs than other imaging methods such as CT scans and ultrasound imaging (8). Since the anatomic cross-lymphatic drainage of the hypopharyngeal region links both sides, contralateral LNM should not be neglected (11). In our group, the rate of bilateral LNM was 16.8%, and all contralateral LNMs were limited to levels II, III, and RPLNs.

Because of the prevalence of LNM in levels II and III, they are generally considered to be the first station for all hypopharyngeal cancers (6, 12), including the preliminary report from our center (13). However, through further we study found that the drainage route varied with different primary sites. In pyriform sinus cancer, almost all N+ patients presented with level II and/or III LNMs. Therefore, we assumed that they were the first drainage stations for PSC. Level IV and RPLNs, with LNM rates following closely behind, were considered as the second station. The third station consisted of Level V and VI because of their rarity. The lymph node drainage diagram (**Figure 2A**) supports our hypothesis. Only 4/143 patients exhibited skipping metastases.

In contrast with PSC, the metastasis rate of RPLN in posterior wall cancer was as high as 38.3%, which was similar to that in levels II and III. Therefore, we assumed that the RPLN along with levels II and III was the first station of LNM for PWCs. In the PSC group, only three patients presented with LNM in Level IV. Therefore, together with Level V and VI, Level IV was also considered to belong to station 2. This hypothesis could also be proved by the simulation diagram (**Figure 2B**), as no skipping metastasis was found.

Another reason why RPLNs were not recognized as the first drainage station before was based on the reports that RPLN metastasis does not appear in N0 patients (6, 14). In other words, pure RPLN metastasis is rare. However, there have been other reports where RPLN metastasis may be present among cN0 HPC patients (15, 16). In our cohort, RPLN metastasis was detected in four patients with negative lateral cervical findings. These results indicate that it is reasonable that the RPLNs are presumed to belong to the 1^st^ station LNMs for PWCs.

According to our finding we suggest that, for cN0 patients with PSC, the elective neck dissection should include level II and III, while for PWC level II, III and RPLNs. When metastasis is considered in the first station LNs, the second station lymph node should be further treated.

The other purpose of this paper is to explore the direction of metastasis of HPC. So we focus on the bilateral and retropharyngeal metastasis, which indicate transfer contralaterally and backward respectively. As a midline organ, the hypopharynx is also drained along the anatomic cross route to the contralateral lymph nodes. Olzowy (17) et al. reported that the incidence of contralateral metastasis was above 20% for HPCs affecting the midline and those involving the medial wall of the PSCs. The overall bilateral LNM rate in our cohort was 16.4%, which is similar to that found in previous reports. Through logistic analysis, we found that HPC with pyriform sinus invasion is more likely to metastasize to the ipsilateral lymph nodes, since pyriform sinus invasion is a protective factor for bilateral LNM. Posterior wall invasion in both univariate and multivariate analyses was proven to be correlated with bilateral LNM, indicating that posterior wall invasion was prone to drainage to the bilateral neck. This phenomenon is easy to explain—posterior wall cancer is located at the midline areas and is prone to drain bilaterally to the neck. However, the pyriform sinus is a lateral structure, and so lymphatic drainage mainly flows in the ipsilateral direction.

The retropharyngeal lymph node has been widely studied in nasopharyngeal cancer and was regarded as the first station for nasopharyngeal lymphatic drainage (14, 18). In recent years, its significance in HPC has received increasing attention (6). Our preliminary study (13, 19) reported RPLN metastasis was related to PWC, posterior wall invasion and cervical LN status. And we found that RPLN metastasis is a poor prognosticator for survival. And in this further study, on multivariate analysis, we found not only posterior wall invasion as a risk factor, but also pyriform sinus invasion was a protective factor for LNM in RPLNs. We could conclude this trend: posterior wall invasion tends to drain back directly to the posterior pharyngeal region, while HPCs with piriform sinus invasion are less likely to drain backward.

Therefore, for radiologists, if pure piriform sinus is invaded, it can be considered that the retropharyngeal area and contralateral neck could not be included in clinical treatment volume (CTV). While, for patients with tumor invading the posterior wall, not only the retropharyngeal area but also both necks should be included in CTV.

Our study was based only on pre-treatment MRI images; thus, occult metastasis could not be evaluated. In fact, due to the wide use of laryngeal conservative strategies, hypopharyngeal cancer mainly adopts radiotherapy-based multidisciplinary treatment. Therefore, it is difficult to admit a large sample of HPCs from the head and neck department. In this study, we obtained a large sample of MRI data from three major cancer hospitals. We believe that this was sufficient for evaluating lymph node metastasis patterns. However, only two cases of PCC were included in the cohort; therefore, more data are needed to understand the metastasis pattern of PCC.

In conclusion, we analyzed 244 pre-treatment MRIs of HPC in three specialized cancer hospitals in mainland China and found that the primary tumor subsite and the extent of invasion were related to the pattern of LNM. PSC tends to metastasize along three stations, while PWC tends to metastasize along two stations. HPCs with piriform invasion were less likely to metastasize to contralateral and retropharyngeal LNs, while posterior wall invasion was a risk factor for bilateral and retropharyngeal LNM.

## Data Availability Statement

The raw data supporting the conclusions of this article will be made available by the authors, without undue reservation.

## Ethics Statement

Written informed consent was obtained from the individual(s) for the publication of any potentially identifiable images or data included in this article.

## Author Contributions

XZ, YZ, and XY contributed equally to this work. XZ statistical analysis and wrote paper. YZ and XY reevaluated the MRIs. CA, YS, and SM collected the patient data. SL, ZL, and JY: supervision. All authors contributed to the article and approved the submitted version.

## Funding

Supported by the Non-profit Central Research Institute Fund of Chinese Academy of Medical Science (2019-RC-HL-004) and Beijing Hope Run Special Fund of Cancer Foundation of China (No. LC2018L06).

## Conflict of Interest

The authors declare that the research was conducted in the absence of any commercial or financial relationships that could be construed as a potential conflict of interest.

## Publisher’s Note

All claims expressed in this article are solely those of the authors and do not necessarily represent those of their affiliated organizations, or those of the publisher, the editors and the reviewers. Any product that may be evaluated in this article, or claim that may be made by its manufacturer, is not guaranteed or endorsed by the publisher.

## References

[1] Carvalho AL Nishimoto IN Califano JA Kowalski LP. Trends in Incidence and Prognosis for Head and Neck Cancer in the United States: A Site-Specific Analysis of the SEER Database. Int J Cancer (2005) 114(5):806–16. doi: 10.1002/ijc.20740 15609302

[2] Newman JR Connolly TM Illing EA Kilgore ML Locher JL Carroll WR. Survival Trends in Hypopharyngeal Cancer: A Population-Based Review. Laryngoscope (2015) 125(3):624–9. doi: 10.1002/lary.24915 25220657

[3] Hall SF Groome PA Irish J O'Sullivan B. The Natural History of Patients With Squamous Cell Carcinoma of the Hypopharynx. Laryngoscope (2008) 118(8):1362–71. doi: 10.1097/MLG.0b013e318173dc4a 18496152

[4] Kotwall C Sako K Razack MS Rao U Bakamjian V Shedd DP. Metastatic Patterns in Squamous Cell Cancer of the Head and Neck. Am J Surg (1987) 154(4):439–42. doi: 10.1016/0002-9610(89)90020-2 3661849

[5] Biau J Lapeyre M Troussier I Budach W Giralt J Grau C. Selection of Lymph Node Target Volumes for Definitive Head and Neck Radiation Therapy: A 2019 Update. Radiother Oncol (2019) 134:1–9. doi: 10.1016/j.radonc.2019.01.018 31005201

[6] Wu Z Deng XY Zeng RF Su Y Gu MF Zhang Y. Analysis of Risk Factors for Retropharyngeal Lymph Node Metastasis in Carcinoma of the Hypopharynx. Head Neck (2013) 35(9):1274–7. doi: 10.1002/hed.23112 22907638

[7] Wu IS Hung GU Chang BL Liu CK Chang TH Lee HS. Is Unenhanced 18F-FDG-PET/CT Better Than Enhanced CT in the Detection of Retropharyngeal Lymph Node Metastasis in Nasopharyngeal Carcinoma? Ear Nose Throat J (2016) 95(4-5):178–84. doi: 10.1177/0145561316095004-506 27140019

[8] Kato H Kanematsu M Watanabe H Mizuta K Aoki M. Metastatic Retropharyngeal Lymph Nodes: Comparison of CT and MR Imaging for Diagnostic Accuracy. Eur J Radiol (2014) 83(7):1157–62. doi: 10.1016/j.ejrad.2014.02.027 24736007

[9] Garneau JC Bakst RL Miles BA. Hypopharyngeal Cancer: A State of the Art Review. Oral Oncol (2018) 86:244–50. doi: 10.1016/j.oraloncology.2018.09.025 30409307

[10] Riviere D Mancini J Santini L Giovanni A Dessi P Fakhry N. Lymph-Node Metastasis Following Total Laryngectomy and Total Pharyngolaryngectomy for Laryngeal and Hypopharyngeal Squamous Cell Carcinoma: Frequency, Distribution and Risk Factors. Eur Ann Otorhinolaryngol Head Neck Dis (2018) 135(3):163–6. doi: 10.1016/j.anorl.2017.11.008 29277379

[11] Mukherji SK Armao D Joshi VM. Cervical Nodal Metastases in Squamous Cell Carcinoma of the Head and Neck: What to Expect. Head Neck (2001) 23(11):995–1005. doi: 10.1002/hed.1144 11754505

[12] Shah JP. Patterns of Cervical Lymph Node Metastasis From Squamous Carcinomas of the Upper Aerodigestive Tract. Am J Surg (1990) 160(4):405–9. doi: 10.1016/S0002-9610(05)80554-9 2221244

[13] Wang H Wu R Huang X Qu Y Wang K Liu Q. The Pattern of Cervical Lymph Node Metastasis and Risk Factors of Retropharyngeal Lymph Node Metastasis Based on Magnetic Resonance Imaging in Different Sites of Hypopharyngeal Carcinoma. Cancer Manag Res (2020) 12:8581–7. doi: 10.2147/CMAR.S245988 PMC7511603 32982450

[14] McLaughlin MP Mendenhall WM Mancuso AA Parsons JT McCarty PJ Cassisi NJ. Retropharyngeal Adenopathy as a Predictor of Outcome in Squamous Cell Carcinoma of the Head and Neck. Head Neck (1995) 17(3):190–8. doi: 10.1002/hed.2880170304 7782203

[15] Amatsu M Mohri M Kinishi M. Significance of Retropharyngeal Node Dissection at Radical Surgery for Carcinoma of the Hypopharynx and Cervical Esophagus. Laryngoscope (2001) 111(6):1099–103. doi: 10.1097/00005537-200106000-00031 11404628

[16] Kamiyama R Saikawa M Kishimoto S. Significance of Retropharyngeal Lymph Node Dissection in Hypopharyngeal Cancer. Jpn J Clin Oncol (2009) 39(10):632–7. doi: 10.1093/jjco/hyp080 19674993

[17] Olzowy B Hillebrand M Harreus U. Frequency of Bilateral Cervical Metastases in Hypopharyngeal Squamous Cell Carcinoma: A Retrospective Analysis of 203 Cases After Bilateral Neck Dissection. Eur Arch Otorhinolaryngol (2017) 274(11):3965–70. doi: 10.1007/s00405-017-4724-3 28840308

[18] Huang L Zhang Y Liu Y Li H Wang S Liang S. Prognostic Value of Retropharyngeal Lymph Node Metastasis Laterality in Nasopharyngeal Carcinoma and a Proposed Modification to the UICC/AJCC N Staging System. Radiother Oncol (2019) 140:90–7. doi: 10.1016/j.radonc.2019.04.024 31195216

[19] An C Sun Y Miao S Yu X Zhang Y Zhang X. Retropharyngeal Lymph Node Metastasis Diagnosed by Magnetic Resonance Imaging in Hypopharyngeal Carcinoma: A Retrospective Analysis From Chinese Multi-Center Data. Front Oncol (2021) 11:649540. doi: 10.3389/fonc.2021.649540 34178636 PMC8226130

